# Experiences of registered nurses and nursing assistants during COVID-19: Work stress, stress appraisal, and workplace resources; A qualitative descriptive study

**DOI:** 10.1371/journal.pone.0345525

**Published:** 2026-03-27

**Authors:** Gillian I. Adynski, Cassandra Dictus, Harry Adynski, Mary K. Killela, Elizabeth Allen Myer, Leah Morgan, Hayden Hmiel, Jessica Williams

**Affiliations:** 1 Duke University School of Nursing, Durham, North Carolina, United States of America; 2 University of North Carolina at Chapel Hill School of Nursing, Chapel Hill, North Carolina, United States of America; 3 National Clinician Scholars Program at Duke University and Durham VA, Durham, North Carolina, United States of America; 4 University of California San Francisco School of Nursing Department of Community Health Systems, San Francisco, California, United States of America; 5 University of Utah: Huntsman Cancer Institute and School of Nursing, Salt Lake City, Utah, United States of America; 6 RTI Health Solutions, Durham, North Carolina, United States of America; 7 Queens University of Charlotte School of Nursing, Charlotte, North Carolina, United States of America; 8 University of North Carolina at Greensboro School of Nursing, Greensboro, North Carolina, United States of America; Universiti Pertahanan Nasional Malaysia, MALAYSIA

## Abstract

The COVID-19 pandemic severely disrupted healthcare systems, placing immense physical, emotional, and organizational strain on front line nursing staff, including registered nurses and nursing assistants. These professionals faced heightened stress due to increased virus exposure, global personal protective equipment (PPE) shortages, and rapidly changing protocols. This study sought to explore the experiences of registered nurses and nursing assistants working in inpatient care during the COVID-19 pandemic, focusing on workplace stressors and available or recommended resources to mitigate these challenges. Despite extensive documentation of elevated stress and burnout among nurses during COVID-19, little is known about how registered nurses and nursing assistants appraised specific workplace stressors and evaluated the adequacy of available organizational resources during the pandemic. Using a qualitative descriptive design, semi-structured interviews were conducted with 14 registered nurses and six nursing assistants from COVID-19 and non COVID units at a large academic medical center. Guided by Lazarus and Folkman’s Stress and Coping Model, the analysis identified a range of personal, interpersonal, organizational, and societal stressors. Personal stressors included long work hours, loss of loved ones to COVID-19, and feelings of isolation. Interpersonal stressors involved exposure risk, emotional strain from coworkers’ stress, and shifts in bedside roles. Organizational stressors encompassed staffing shortages, changes in protocols, and being called off shifts. Societal stressors included inconsistent public health messaging and concerns about protecting vulnerable family members. Participants emphasized the importance of authentic leadership and nursing-centered delivery of resources in addressing these stressors. Six key resource categories emerged: emotional support, staffing, safety, compensation, communication, and stress management. Findings highlight the critical role of nurse managers, effective communication, and staffing policies in mitigating workplace challenges. While rooted in a U.S. context, these insights may inform strategies to support nursing staff globally in future crises, reinforcing the need for tailored, sustainable approaches to front line caregiver well-being.

## Introduction

The COVID-19 pandemic created unprecedented challenges for healthcare systems, particularly for registered nurses (RNs) and nursing assistants (NAs) on the frontlines. Coronavirus disease 2019 (COVID-19) is a highly infectious respiratory illness caused by the severe acute respiratory syndrome coronavirus 2 (SARS-CoV-2), which led to a global pandemic beginning in late 2019. These professionals faced significant changes to patient populations and treatments, work environments, and daily workflows, which can contribute to heightened stress levels [1]. RNs and NAs were at high risk of experiencing stress due to their critical role in patient care during the pandemic and the rapid changes to their daily work likely increased work-related stress, regardless of caring for COVID-19 positive patients [1]. Significant global Personal protective equipment (PPE), including items such as masks, gowns, gloves, and face shields, shortages across high- and low-income countries left healthcare workers lacking standard PPE and reusing single-use items, leading to adverse effects like heat, thirst, pressure sores, headaches, bathroom access issues, and extreme exhaustion [2]. Nursing staff were at increased risk for COVID-19 due to their direct exposure to airborne and droplet transmission, as they spent more time in patient rooms than other healthcare professionals even before the increased demands of the pandemic [3]. This prolonged exposure translated into higher infection and mortality rates among RNs than physicians during the pandemic [4]. In addition to infection and mortality disparities, descriptive studies have shown an increased level of anxiety, depression, and insomnia for healthcare workers during COVID-19 [5–7]. A study from the end of 2020 showed almost 70% of RNs reporting anxiety symptoms with almost 40% being in the moderate and severe range [8].

Stress and anxiety in the healthcare workplace can have profound consequences, including impacts on patient and organizational outcomes. Workplace stressors include increasing demands and decreased resources are directly linked to burnout [9–11]. Burnout, defined as emotional exhaustion, depersonalization and lack of personal achievement at work, [12] has detrimental effects on both individuals and healthcare systems. RN burnout leads to lower patient satisfaction and lower quality of care [13]. High burnout levels and low resilience lead to increasing turnover of the nursing workforce [14–17], resulting in significant financial costs for organizations and the loss of valuable institutional knowledge held by departing staff [18]. In the United States (U.S.) turnover can cost range between $22,000 to over $88,000 per RN [19].

Although a substantial body of literature has examined RN stress during the COVID-19 pandemic, nursing assistants’ experiences remain underrepresented, particularly within studies examining how inpatient nursing staff appraised workplace specific stressors and available resources [20]. NAs are often left out of the literature even though their responsibilities to provide personal patient care place them in close proximity to the stressors of caring for patients during COVID-19, just like their nurse counterparts. Addressing these gaps is critical for guiding healthcare leaders, researchers, and policymakers as they prioritize efforts to promote resilience and reduce burnout among the current nursing workforce, who are still experiencing residual stress of COVID-19. Understanding the experiences of inpatient RNs and NAs during the pandemic, in their own words, allows for the identification of actionable strategies to address stress and burnout both during future crises and in routine practice. These insights may be applied to other non-inpatient settings, [21,22] as the challenges faced by nursing staff are reflected across diverse healthcare contexts globally.

This qualitative descriptive study, guided by Lazarus and Folkman’s Stress and Coping Model, [23,24] aimed to explore the experiences of RNs and NAs in the southeastern U.S. providing inpatient care during the COVID-19 pandemic. The present study focuses on exploring how nursing staff experience work-related stressors, appraisal of that stress, and the workplace resources that were available or recommended to manage these stressors. Although Lazarus and Folkman’s framework encompasses both individual coping processes and environmental factors, this study deliberately focuses on organizational stressors and structural resources, and does not examine individual-level coping strategies such as self-care or professional resilience. This work addresses critical gaps in our understanding of how RNs and NAs perceive similar events as stressful or not, and the workplace resources they identify to cope with work-related stress, particularly during heightened periods such as the COVID-19 pandemic. Although centered within U.S. context, these findings and recommendations can be relevant internationally, especially in inpatient care settings where nursing staff faced similar challenges during the pandemic. These findings can also be applied to other crisis situations, offering valuable insights for supporting healthcare workers in times of extreme stress.

## Materials and methods

### Design

This study employed a qualitative descriptive design to explore the experiences of inpatient nursing staff during the COVID-19 pandemic. Data were collected through semi-structured, individual interviews with registered nurses (RNs) and nursing assistants (NAs) who were providing direct inpatient nursing care during the pandemic. This approach allowed for consistency across interviews while maintaining flexibility to probe individual experiences related to workplace stressors, and available resources. A key strength that supports rich, practice-relevant descriptions grounded in participants’ own words. A limitation of this design is that findings reflect experiences within a specific institutional context; however, detailed reporting of the setting and analytic process supports the trustworthiness and applicability of the findings to similar care settings. The study adhered to the COREQ (Consolidated Criteria for Reporting Qualitative Research) guidelines to ensure standardized and transparent reporting of qualitative research methods and findings [25] (see S1 Appendix file).

### Theoretical framework

The study was guided by Lazarus and Folkman’s Stress and Coping Model, [23,24] which conceptualizes stress as a process involving primary appraisal (evaluating whether a stressor is perceived as a threat or not, with positive appraisals indicating no threat and negative appraisals indicating a threat) and secondary appraisal (assessing the availability of internal and external coping resources, with positive appraisals indicating resources are available and negative appraisals indicating they are not). Utilizing this framework, directed content analysis was applied to inform the development of the interview guide and shape the qualitative analysis. Qualitative, semi-structured interviews examined how the COVID-19 pandemic affected the work experiences of RNs and NAs, their perceptions of job-related stress, the availability of workplace resources, and recommendations for future resources to address healthcare system crises. The interviews also explored how RNs and NAs appraised and managed stressors during the pandemic, with particular attention to the effectiveness or absence of workplace resources (See S2 Appendix for Interview Guide). The interview guide was piloted with a nurse, RN not employed at the medical center, to ensure clarity, relevance, and alignment with the study objectives.

### Study setting, sample, and recruitment

This project was conducted at a large academic medical center in the southeastern U.S. A purposeful stratified sampling method was used to gather representation of both COVID and non-COVID inpatient nursing staff to have a comprehensive view of the experience of working during a time of crisis. Target sample sizes were set at 6 participants across RN-COVID, RN-Non-COVID, NA-COVID, and NA-Non-COVID to achieve balanced representation across these groups. Participants were recruited through informational flyers posted throughout the hospital, hospital-wide employee listservs, and in-person recruitment at individual unit staff meetings.

Recruitment continued until predetermined recruitment goals were met. Despite repeated recruitment efforts, we were unable to meet the target enrollment goals within the NA-COVID subgroup. We did not assess saturation within subgroups because the analysis was not designed to compare experiences across subgroups; rather, purposeful subsampling was used to ensure a range of experiences, including those of underrepresented nursing assistants, were reflected in the data. A final sample of 20 participants is consistent with qualitative research standards for data sufficiency [26]. During analysis, code saturation was assessed, defined as the point at which no new codes were identified in successive transcripts [26]. No new codes emerged in the final transcripts, supporting that the sample size was sufficient to address the study aims.

### Inclusion and exclusion criteria

Inclusion criteria included full-time RNs or NAs who spent >80% of their clinical time delivering direct patient care at the bedside and had worked on their current unit for at least three months before the start of the pandemic. COVID units were defined as floors that primarily take care of patients with COVID-19 (e.g., COVID-19 intensive care units, COVID-19 acute care units, Emergency Department (ED)). Non-COVID units included all other inpatient units within the medical center (Medical Surgical, Step Down, Women’s, Pediatrics, Psychiatry). Stratification across hospital roles and unit type was used to ensure diversity of experience, capturing perspectives from nursing staff working in both COVID-designated and non-COVID units, as well as different clinical settings and patient populations.

### Data collection

Participants completed initial consent as a part of an initial eligibility screening survey via Qualtrics where they learned about the goals for the proposed research. During recruitment participants were informed they would be compensated for their participation in the qualitative interview. A total of 71 participants expressed interest in completing an interview, including 58 registered nurses (9 RN-COVID, 49 RN-Not COVID) and 13 nursing assistants (0 CNA-COVID, 13 CNA-Not COVID). Participants were then enrolled on a first come, first serve basis. If capacity was reached for a certain sampling stratification, each subsequent participant was placed on a waitlist to be contacted if attrition occurred. If a participant was unable to be contacted after three failed attempts, the next eligible individual on the waitlist was contacted. Eligible individuals received a confirmation email to schedule a 45–60 minute Zoom Interview. Within the scheduling email, additional mental health resources were provided, including a copy of the informed consent document. At the beginning of the interview, the study team reviewed the consent information with participants and obtained verbal consent before proceeding with the interview. Participants completed the Zoom interviews in private settings, such as their homes or private offices, to ensure confidentiality and minimize potential interruptions. Participants were allowed to stop the interview at any time for any reason; however, no participants chose to withdraw from the study or discontinue the interview or required a repeat interview. Interviews were conducted by trained study team members (MK, EM, CD, HH). All interviews were audio-recorded and professionally transcribed verbatim. Research team members checked all transcripts for accuracy against the audio-recordings and de-identified the transcripts to protect participant confidentiality. After the interview, the participants received a $50 gift card by email as compensation for their time. Trained interviewers also completed a brief memo to document their impressions of the interview and to engage in reflective practice. Interviews were conducted between April 2021 and September 2021.

### Data analysis

All interviews were analyzed using directed content analysis with deductive coding and theming, guided by the Stress and Coping Model, while also allowing for emergent themes to arise [27,28]. Dedoose (Los Angeles, CA) qualitative software was utilized to code all de-identified interviews. Initially, the codebook was generated based on the Stress and Coping Theory. The initial codebook was piloted on a subset of interviews and definitions were refined and additional codes were added.

Three teams of two coders independently coded each interview (CD, HA, MK, EM, LM, HH), reviewed the interview notes, and subsequently convened to reach a consensus. Two coders worked independently to code the interviews and then met to reconcile codes and review memos [29]. To ensure dependability, an audit trail documenting all work, including raw data, data synthesis, and process notes, was maintained throughout the process [30]. We developed a hierarchical coding tree grounded in the Stress and Coping framework, comprising major domains of stressful events, primary appraisal, secondary appraisal, adaptive outcomes, and resources (including resources offered or available to participants and recommended resources), with each domain subdivided into conceptually related child codes. These teams then met with the larger group of coders to further debrief and refine the codebook as new themes and subthemes emerged through an iterative process. This procedure was conducted for 14 interviews. Once the codebook reached stability, the remaining 6 interviews were coded independently until data saturation was reached [31].

### Ethical considerations

Ethical approval to conduct this study was obtained by the institutional review board at the University of North Carolina at Chapel Hill (#20–2323) as well as approval from the hospital governance nursing research council within the medical center.

### Rigor and reflexivity

To enhance the rigor of the study, credibility was ensured through investigator triangulation, where multiple coders analyzed the data to validate findings. Additionally, an audit trail was maintained, including reflective memos and documentation of analytic decisions, to promote transparency and dependability.

Reflexivity was integral to the research process, as all members of the research team including coders, study designers, and authors shared professional nursing backgrounds, with many actively working in inpatient settings during the COVID-19 pandemic. The research team includes six PhD-prepared RN (PhD-RNs) and one bachelor’s-prepared RN who is currently a Doctor of Nursing Practice student. The research team comprised five female members, two male members, and one gender-expansive member, reflecting diverse gender representation. At the time of data collection among the PhD-RNs, four were PhD students, four were employed as registered nurses within the healthcare system where the data was collected, one was a faculty member at a university, one a post-doctoral fellow, and one was a BSN nursing student. Importantly, none of the team members had established personal or professional relationships with the participants, nor did they hold supervisory or hierarchical roles over them, reducing the potential for bias. Participants were informed that the interviewers were registered nurses. The research team’s identities as nurses and firsthand experiences allowed them to bring valuable insights into the nuances of nursing practice and challenges faced during the pandemic, while also shaping the framing of the research questions and interpretation of data. The research team brought extensive experience and training in nursing practice, healthcare systems, qualitative and quantitative research methods, and public health. Their expertise included clinical practice as registered nurses, advanced academic preparation in doctoral programs, faculty roles in higher education, and specialized knowledge in areas such as mental health, community health, and healthcare policy. Recognizing the potential for these shared experiences to introduce bias, the team engaged in deliberate reflexive practices, such as regular discussions about positionality, reviewing reflective memos to document assumptions and insights, and holding team meetings to critically examine the coding process. These strategies ensured that the findings were firmly grounded in the data and authentically represented participants’ voices, rather than being overly influenced by the researchers’ personal experiences.

## Results

### Characteristics of participants

The final sample included 20 participants, 14 of which were RNs and 6 of which were NAs. Of the RNs, 1 had a nursing diploma, 4 had associate’s degrees, 8 had bachelor’s degrees, and one had a master’s degree. At the hospital from which the sample was collected, 13 participants worked on units that accepted and treated patients who were known to be COVID + , and 7 participants worked on units that did not accept known COVID+ patients. All participants were recruited from the same large academic medical center in the southeastern United States. Table 1 offers a demographic overview of all participants.

**Table 1:** Participant demographic data.

	Mean or n	sd or %
**Age**	35.9	9
**Gender**		
Male/Man	2	10
Female/Woman	18	90
**Race**		
White	13	65
Black/African American	5	25
Native American	1	5
Multiple	1	5
**Ethnicity**		
Hispanic/Latiné/Latinx	1	5
Non- Hispanic/Latiné/Latinx	19	95
**Role**		
CNA	6	30
RN	14	70
**Education**		
NA I Certification	5	25
NA II Certification	1	5
Diploma in Nursing	1	5
ADN	4	20
BSN	8	40
MSN	1	2
**Unit Type**		
Medical Surgical	6	30
Step Down	3	15
Intensive Care Unit	5	25
Women’s	2	10
Pediatrics	1	5
Psychiatry	3	15

### Main findings

This paper aimed to define nursing workplace stressors and resources along with the various positive and negative ways RNs and NAs appraised these stressors and resources. The main findings of this paper will be divided into two sections of stressor and resources. RN and NA stressors, definitions, and primary appraisals, and quotes are illustrated in Table 2. Resources, definitions, secondary appraisals, and quotes are illustrated in Table 3.

**Table 2:** Registered nurses and nursing assistant stressors, definitions, and primary appraisals.

Stressor	Definition	Primary Appraisal of Stressor	Exemplar Quote (with appraisal underlined and bolded)
**Personal stressors**	External stressors outside of work that significantly impacted participants’ professional lives during the pandemic. • Financial stress or reduced work hours. • Changing childcare needs. • Feelings of isolation. • Loss of loved ones to COVID-19 or other ailments. • Working extra hours due to COVID demands. • Being out of work or facing reduced hours due to decreased elective procedures.	**Threat/ Negative**	“One of the biggest stressors for me has been the management of my family during all of this. That has been more [hard] on me than the actual job has. When it first started, I sent my kids away for two months, and they didn’t understand. Of course, I’m not telling them the things that I’m dealing with because I don’t want to scare them. When they came back, I was **afraid** to even hug them.” *-COVID Unit RN 2*
		**Non-Threat/** **Positive**	“I would say that the COVID patients are, we did notice, **easier** compared to, you know, the general population we were getting earlier… They would come in with their specific needs, like the oxygen or if they had GI issues or neuro issues. But besides that, it was more straightforward with the COVID patients versus our other patients that were more complex. So in terms of that, it’s actually better. **We actually prefer the** **COVID** **patients.** Um, but yeah, I would say it’s, it’s about the same. [for] at least me personally, I didn’t feel like it’s a big difference in terms of, like, my nursing care. It’s just, you know, in the beginning it was more stressful, um, just because of, like, having to stay on top of all of the new policies. *-COVID Unit RN 4*
**Interpersonal stressors- coworkers**	Challenges stemming from difficulties in achieving harmony and collaboration among coworkers. • Frustration over bedside role expectations and unequal risk exposure during COVID-19. • Communication breakdowns or lack of teamwork. • Bullying or conflicts among peers. • Vaccine hesitancy or reluctance to care for COVID patients. • Difficulties obtaining consults or support. • Experiencing or witnessing racism in the workplace. • Emotional strain from observing coworkers under significant stress.	**Threat/** **Negative**	“Sometimes, I would see some of the aides, cracking the door asking for something. *[However],* if another aide was on the floor and I asked them to, just push it through the door so I don’t have to de-robe, come out of the room, and then do it all over again. They’d be like, “No, I don’t want to come near…” So they’d make my job a lot harder. One day, I must’ve donned, and, and doffed about, probably close to 20 times” *-Non-COVID Unit CNA 8*
		**Non-Threat/ Positive**	“But just work support, kind of knowing that people had your back, that you weren’t going to be taking care of the patients alone. There were certain people that had such good morale. Even if they were faking it, maybe they were miserable inside, but I’d say support at the job from coworkers on certain units was great. can’t say enough about those nurses, um, on those units.” *-COVID Unit RN 8*
**Interpersonal Stressors- Patients and Families**	Stress caused by challenging interactions with patients and patients’ families, heightened during the COVID-19 pandemic. • Risk of exposure to the virus. • Dealing with argumentative or combative patients. • Caring for higher-acuity patients than usual. • Coping with patient deaths.	**Threat/ Negative**	“I had more patients die in the last 18 months, my personal patients, than I had in 20 years combined. And I’ve been an ICU nurse for 20 years, so that’s a lot of death... **Grief** is not something that goes away. It’s compounded. So, every time you have an event like this, it just sits... it settles as a layer on top of the last bit of grief you have.” *-COVID Unit RN 9*
		**Non-Threat/ Positive**	“Just that extra few minutes in there, that made my day. That made me feel like I was doing something positive even though the work environment may not be feasible or like we might feel like no one’s really listening to us. But the fact that you know that you’re making a difference in your patient’s life, like you’re really the one that’s making sure they get enough oxygen, making sure they’re comfortable, you know. So, I think that was, for me, that was really the biggest impact for why I worked with the COVID patients. -*COVID Unit RN 1*
**Organizational stressors**	Increased responsibilities and workplace changes that create stress and alter the work environment. • Expectations and pressures from leadership. • Changes in work protocols or uncertainty around PPE usage. • Staffing challenges, such as team departures or integrating traveling staff. • Taking on charge nurse duties or managing unfamiliar patients. • Being called off from scheduled shifts.	**Threat/** **Negative**	“I remember the biggest **fear** and what I know will probably be the biggest hype of our interview was the **fear** around floating off of service. And that that was the hot topic. And what was really happening was nurses throughout [*our hospital system]* and not just at our campus, but all over were being floated outside of our scope. I’ve never taken care of a male patient [*non-postpartum*] and just feeling totally out of my wheelhouse.” *- Non COVID Unit RN 14*
		**Non-Threat/ Positive**	“I think at the beginning, it was exciting and new because you... I mean, maybe not for a new nurse, but I had been a nurse for a while, so it was exciting and new because you thought you could finally use your skills and help.” *-COVID Unit RN 8*
**Societal stressors**	Stress related to societal-level challenges and responses during the COVID-19 pandemic. • Lack of accurate or consistent public information on COVID-19. • Concern for protecting immunocompromised individuals. • Fear of premature public reopening causing hospital surges. • Worry about personal or family exposure to COVID-19 in public spaces.	**Threat/** **Negative**	“At first it was, it felt overwhelming. It felt **scary.** Obviously, not having worked through something like this, I just didn’t know how to feel. And I just knew that I was feeling a lot of anger towards others when it came to not wearing a mask or not taking it seriously.” *-Non-COVID Unit CNA 9*
		**Non-Threat/ Positive**	“When you need to go to summer camp and you have to swab yourself or swab your kids to go, at this point, it almost seems sort of the new norm. Where a little over a year ago, it was super scary.” *-Non COVID Unit RN 15*

**Table 3:** Resources, definitions, appraisals and quotes.

Resource	Definition	Resource (secondary) Appraisal	Quotes (with cross-cutting themes of authenticity and nurse centeredness underlined and bolded)
**Emotional support from managers and coworkers**	Emotional support from managers, peers, and chaplains, essential for RNs and NAs during the pandemic. • Managers checking in through individual or group meetings. • Chaplains providing avenues to decompress and find solace. • Emotional support from coworkers at work and in personal lives. • Disparities in access to emotional support, highlighting its critical role in well-being and resilience.	**Negative**	“From a more management aspect of it, they aren’t as personal and, especially when I was in the float pool because we have such a huge staff it’s hard to be on one and personal with staff. I don’t think they were as helpful.” *-Non COVID Unit CNA 7*
		**Positive**	“My coworkers are great, they’re my second family. We’ve had crying sessions together; my unit is very supportive. I feel like we were able to keep each other afloat.” *-Non COVID Unit CNA 4* “On a unit-based level [it would be helpful] to have check-ins from the chaplain more regularly. Because a lot of times there is this sort of mentality of like if you stick it out long enough versus like it [support] being there and being offered to you and you kind of walk in and say, ‘Oh, okay. You know, I’ll take you up on that.’ The chaplains are incredible at like taking a situation that seems untenable and saying, “You know, all right, but you’re doing really well in this situation” *-Non COVID Unit RN 7*
**Staffing and personnel resources**	Staffing and retention resources that eased the stress of providing care during COVID-19. • Improved staff-to-patient ratios. • Travel RNs filling staffing gaps. • Support from experienced staff members. • Retention efforts, such as stay bonuses, instead of high/signing bonuses for travel nurses.	**Negative**	“They could have just hired more assistants. One night I had the choice of either holding pressure on someone’s chest so that they didn’t bleed from their chest tube anymore, and I was just to hold pressure there for 20 minutes, but my other patient needed a urinary catheter put in because they had like 600 ml a urine in their bladder, and I was charge and have three patients. So, I at one point I had to have an aid, hold pressure for me while I went to go put in a urinary catheter. I said, ‘you just need to hold your hands here for 15 minutes until I come back’, and I had the first person tell me, no. Then the second person reluctantly did it. I don’t know what other choice I had. There was nobody else there to step in.” *-Non COVID Unit RN 6*
		**Positive**	“I wish they had dealt with the staffing situation better. They waited a really long time to hire travel nurses and get more staff on board. And I think we were struggling with some, not quite, but almost unsafe patient nurse ratios. And we had to work really way harder than we should have to make it as safe as it was.” *-Non COVID Unit RN 5*
**Personal protective equipment and safety resources**	Safety involves resources, processes, or systems that protect RNs from harm in their professional duties. • Availability of adequate personal protective equipment (PPE). • Adherence to infection control policies, such as vaccination mandates and symptom checks. • Access to security personnel. • PPE and vaccine-related concerns as key safety issues.	**Negative**	“We thought we’re going to catch COVID at work because we would get these patients that would have to be intubated in a room or were coughing all over the place and kept taking their masks off. We just felt it was inevitable. It’s just a matter of time before I catch it. These goggles that I’m supposed to be wearing, I feel it’s a false sense of security” -Non COVID Unit RN 6
		**Positive**	“Over time, **we got more comfortable, and we realized that we were doing the right thing and that we had the right protection.** We had enough protection and enough PPE, so I think our confidence just grew, and we got more comfortable with our new norm. And then I could tell you that what made us ultimately feel better was the talk of the vaccine and then ultimately getting vaccinated.” *-Non COVID Unit RN 15*
**Benefits, compensation, and gifts**	Financial incentives and gestures of appreciation that supported and motivated RNs during the pandemic. • Hazard pay, tuition reimbursement, and parking assistance. • Accessible childcare and benefits like PTO, FMLA, and disability support. • Community and workplace tokens of appreciation, such as cards, food, and parties. • Recognition events like ice cream or pizza parties.	**Negative**	“What are you doing here with ice cream? Like, that’s not doing anything here in the trenches. It’s just like a slap to our face. We’ll just give them ice cream and just shut them up. They’ll be fine. No! I felt like we were going through so much mentally, physically…and they claimed they tried? I felt like they were not really listening to us.” *-COVID Unit RN 1*
		**Positive**	“They started paying us really well for overtime shifts. So, I’m working extra shifts. It helps but it’s still exhausting (laughs). I’ll take advantage of it while it’s here. I know a lot of nurses were really hoping we would get hazard pay or maybe some student loans paid off but it’s cool. I’ll take what I can get.” *-Non COVID Unit RN 5*
**Communication and knowledge resources**	The need for up-to-date information and effective communication channels to navigate the evolving challenges of COVID-19. • Rapidly changing protocols and knowledge due to the emerging nature of COVID-19. • Communication channels like town hall meetings, training sessions, and journal clubs. • Access to the intranet, huddles, resource nurses, and routine check-ins for necessary information.	**Negative**	“[I recommend] open forums or town halls of just letting people talk and bring about their concerns or questions, just more transparency. I felt like there was many times where we didn’t really know how to move forward or what to do, and we really had to reach out, and we still are, as far as, COVID testing our patients, or what does that mean, or what kind of tests do our patients need to have, and what do we need to be wearing. When you get information and then it suddenly changes, it was hard in the beginning to really have that trust, between us as workers and our place of employment as to, “Well, are you really sure this is what we’re supposed to do?” Um, and are we really safe?” -Non COVID Unit RN 15
		**Positive**	“Every week, our manager would send us the email, Webex, so we were informed pretty much what’s going on everywhere, what we have to follow [regarding PPE protocols].” *-Non COVID Unit RN 10*
**Health, wellbeing, and stress management**	Health, well-being, and stress management resources provided by hospitals to support nurses during stressful times. • On-site cafeterias and coffee availability. • Conference rooms for lunch and break/quiet rooms. • Pet therapy and access to TVs. • Support groups and Employee Assistance Programs (EAP). • Wellbeing indexes, online wellness resources, and meditation options. • Encouragement of physical activity to manage stress and promote mental health.	**Negative**	“There’s all this talk about self-care and wellness, but if the organization refuses to allow you the time to perform basic care, like drinking water, going to the bathroom and eating food, if everything stands in the way of that, then [self-care initiatives are] just like a face value thing, it’s not real.” -Non COVID Unit RN 6
		**Positive**	“My hospital offered counseling where you get a couple of free sessions. So, I went for the sessions, and I realized it was helping me, it was helping the mental aspect of being in the pandemic and working in the hospital. So, I decided to keep going.” -Non COVID Unit CNA 8

### Stressors

The major types of stressors that RNs and NAs experienced can be categorized as 1) personal stressors, 2) interpersonal stressors- coworkers, 3) interpersonal stressors- patients and patients’ families, 4) organizational stressors, and 5) societal stressors. The next section will go over the definitions of these stressors and examples of RNs’ negative and positive appraisals of them. Table 2 outlines the stressors identified by RNs and NAs, including definitions, primary appraisals, and illustrative quotes.

#### Personal stressors.

Participants described several stressful events that occurred outside of work, that impacted their work lives, such as financial stress, changing childcare needs, feelings of isolation, and loss of loved ones to COVID-19 or other ailments. While some RNs were expected to work extra hours due to COVID-19, many were asked to reduce their hours because their units canceled scheduled shifts due to low census. (e.g., canceled elective procedures in the early stages of the pandemic). This reduction in work hours led to some nurses to experience increased financial distress from working fewer hours.

Many RNs endorsed general distress from their personal stressors including feelings of helplessness, fear, exhaustion, anger, anxiety, overwhelmed feelings, and feeling of being on an “emotional roller-coaster”.

Few participants perceived the COVID-19 pandemic as non-stressful or reported feeling indifferent to changes in their personal stress levels. When participants described positive or neutral stress appraisals, they described additional context as to why they felt this way. Some noted that other personal stressors such as the death of a family member unrelated to COVID-19 overshadowed the challenges of care delivery during the pandemic. Some staff members described caring for COVID-19-positive patients as less stressful than other patient care. Once policies and treatment protocols were established, the care became more standardized, which made it more familiar compared to patients with more unknown and complex care needs. Many described the familiarity of working with COVID positive patients as ‘the new normal’. Similarly, others shared that previous clinical experience with infectious disease care helped them feel more confident and comfortable providing care during this time.

#### Interpersonal stressors- coworkers.

These were challenges that arose from heightened difficulty in achieving harmony between and among coworkers. This included communication breakdowns, bullying, or vaccine hesitancy among staff. Specific stressors involved the transition to becoming a COVID unit, bullying from peers, reluctance among staff to care for COVID-19 patients, and the need for stronger teamwork and communication. Additional factors included difficulties in obtaining consults, experiencing or witnessing racism at work, and the emotional toll of watching coworkers undergo significant stress.

Negative appraisal of interpersonal stress involved viewing stressors related to interactions with colleagues and support systems as harmful, worsening feelings of isolation, burnout, and lack of support. Participants expressed feeling abandoned when coworkers avoid COVID-19 rooms, increasing their workload and intensifying isolation and resentment. The burnout of colleagues further strained the remaining staff, heightening stress and frustration. Participants discussed frequent absenteeism exacerbated staffing shortages, overwhelming those present. Furthermore, participants stated insufficient management support can make RNs feel undervalued and unsupported. Lastly, non-compliance with safety protocols was perceived to increase risk and mistrust among staff.

Participants also reported feeling frustrated about their role at the bedside and the assumptions surrounding their work hours. During the COVID-19 pandemic, they were required to have direct patient contact and spend long hours in COVID-19 rooms, while some of their colleagues avoided entering patient rooms and opted for telemedicine care. This often meant that nursing staff had to enter the room an additional time just to deliver telemedicine equipment, adding to their workload and exposing them to risk to accommodate a colleague.

Positive appraisal of interpersonal stress involved viewing stressors related to interactions with colleagues as opportunities for growth, solidarity, and pride in the profession. Some participants described their peers as heroes, which instilled a sense of pride and reinforced their commitment to nursing. Reliance on coworkers for support with PPE and patient care strengthened teamwork, making stressful situations more manageable. Additionally, even when peers masked their true feelings, positive attitudes helped uplift individual spirits and foster a supportive environment. For example, one participant described how she knew her colleague was “faking it,” but the positive outlook helped her regardless.

#### Interpersonal stressors- patients and families.

Participants stated being under immense stress due to their interactions with patients and their families, especially during the early stages of the COVID-19 pandemic. The risk of exposure to the virus, dealing with argumentative or combative patients, caring for higher-than-normal acuity or different patients, and coping with patient deaths all contributed to the described heightened stress levels.

Participants’ emotional reactions to the stressors of COVID-19 patients were diverse. Participants expressed fear and stress surrounding conflicts with patients and their families, often stemming from restricted visiting hours for patients’ families. Participants expressed stress related to the increased acuity of patients causing higher and more challenging workloads. Finally, participants expressed immense feelings of grief and sadness related to the increased death and dying of patients.

Participants had some strong positive appraisals of caring for patients as well, and these positive appraisals largely stemmed from feeling as though they were making a difference in the lives of their patients and their families, leading to a rewarding feeling. Others reported that COVID-19 patients’ care was not inherently more complicated than their pre-pandemic usual patients and did not feel significantly different in that regard.

#### Organizational stressors.

Organizational stressors were defined as increased responsibilities and/or challenging processes that alter the work environment. They arose from expectations and pressures from leadership, changes in work protocols, uncertainty surrounding PPE usage, and shifts in staffing, such as team members leaving, the integration of traveling staff, or the dynamics of interdisciplinary teams. Additional stressors included assuming charge RN responsibilities, fluctuations in workload, caring for unfamiliar patients, and shortages of essential supplies like medications. Other factors contributing to stress included lack of access to food and coffee during off-hours, being called off from scheduled shifts, and navigating a changed work environment despite no formal changes to job requirements.

Participants expressed feelings of loss of control and frustration with power dynamics, where power struggles and breakdowns in communication led to feelings of not being listened to or valued. RNs and NAs described feeling less confident in their skills and feeling used, dehumanized, or expendable. RNs also described how increased workloads, time spent in patient rooms, and assuming the risk of COVID-19 exposure were expected of nursing staff more than other healthcare workers. For example, one RN described needing to enter a patient’s room additional times to set up a video conference call with a physician so that the physician team would not need to enter the room.

Alternatively, some participants expressed feelings of being valued and grateful for their jobs. They successfully adapted to new workflows and found confidence in their abilities, which made the situation more manageable, along with proper PPE. Improvements in the work environment, including staffing changes, contributed to reduced stress. While many recognized both the organizational challenges and benefits, some participants reported a growing sense of progress, with stress levels decreasing and the acceptance of the “new normal” becoming routine. A clearer understanding of expectations provided reassurance.

#### Societal stressors.

Participants felt stress at the societal level stemming from the universal experience of COVID-19 as a stressor at a local, state, national, and international level. Participants expressed concern regarding a lack of available information at a societal level on COVID-19, varied community responses (or lack thereof) to COVID-19, and a concern about protecting the immunocompromised in society at large. Participants were concerned about relaxing distancing guidelines too early that could cause surges in cases at hospitals. Lastly, participants discussed fearing for their family members and themselves being exposed to COVID-19 in public.

Participants described societal stress from fear of acquiring COVID-19 due to a lack of clear national safety guidelines, as well as fear of premature relaxation of distancing guidelines and rising COVID-19 infection rates. Participants described specific fears about immunosuppressed loved ones suffering from government public health mandates that could put vulnerable populations at risk. Participants expressed that the beginning of the pandemic was a time of heightened fear and uncertainty, yet these feelings decreased over time.

While few participants stated truly positive appraisals of the societal level stressor of COVID-19, many expressed a quick adjustment and acceptance of COVID-19 as the new normal. They adapted to testing for infection before attending events and helping their families navigate this space. Others expressed how they could handle the stressor of the pandemic as long as they had the proper PPE.

### Resources

Two overarching themes related to authenticity and nurse-centeredness in the delivery of resources were identified, along with six types of resources: emotional support, staffing, safety, compensation, communication, and stress management. Both positive and negative appraisals were observed across all resource types. Table 3 presents these resources, along with their definitions and the positive and negative evaluations from RNs and NAs for each resource.

#### Authenticity and nurse-centeredness in the delivery of resources.

Two cross-cutting themes emerged regarding the delivery of resources. Across many different types of resources, participants emphasized the importance of how resources were delivered. They described key themes such as authenticity, individualization, and integration of supports into their workflow. For example, participants noted that the same small gesture could evoke drastically different reactions depending on the context of its delivery. A large ice cream party from hospital administrators was perceived as “a slap in the face” or dismissive and impersonal, whereas a chaplain offering a cookie during a one-on-one visit was perceived as genuine and supportive. Participants thought it was important that there were a variety of resources offered, recognizing that what works for one person might not be the same as what helps someone else. Participants also discussed barriers to accessing and utilizing certain resources, which often felt like “lip service” or superficial efforts rather than meaningful support. They expressed frustration that if management genuinely valued their mental health, they would prioritize structural changes, such as providing protected time for lunch breaks.

#### Emotional support from managers, chaplains, and coworkers.

Participants noted the importance of emotional support from managers and coworkers as a crucial resource for RNs and NAs navigating the challenges posed by the pandemic. Requests for emotional support encompassed various forms of support, including managers proactively checking in on nursing staff through individual or group meetings, chaplains offering avenues for RNs to decompress and seek solace amidst the distressing experiences encountered at work, and peers extending emotional support to one another, both within the workplace and in their personal lives. Many participants viewed this support positively, highlighting that their managers did what they could within the constraints of the crisis situation, and chaplain services were especially appreciated, with some recommending expanded access to chaplain support. Peers were also recognized as a vital source of strength due to their shared experiences and mutual understanding. However, not all participants experienced this support. Some described a lack of emotional support from both peers and management, especially among those working in float pool positions who were not assigned to a consistent unit. These participants often felt isolated and overlooked, emphasizing the uneven distribution of support and its impact on staff well-being. This contrast underscores the critical role that consistent emotional support plays in bolstering resilience and sustaining the healthcare workforce during crises.

#### Staffing and personnel.

Participants endorsed good staff-to-patient ratios, with travel RNs to meet gaps and experienced staff to better manage the stress of providing care during COVID-19. Retention efforts such as hazard pay or bonuses for staying rather than paying travel RNs high/signing bonuses was a highly requested intervention.

Staffing was typically the first area mentioned by participants when asked about recommended supports. Participants noted challenges around short staffing and the way staffing was handled. Participants described being floated to unfamiliar units to meet staffing demands, which was particularly challenging especially if being floated to a different nursing specialty. Alternatively, some reported a reduction in work hours due to changes in providing elective procedures and a decrease in patient volume census. Participants recommended a greater emphasis on retention (bonuses for staying rather than or in addition to hiring bonuses), noting the importance of maintaining experienced staff given many left the institution for example to retire early or seek better paying travel nurse contracts. They also mentioned challenges because of shortages in other roles of the health care team and the importance of the larger collaborative team, as many tasks fell to RNs to do. For example, due to limited hospital visitors and reductions in support staff had to take on responsibilities that would typically be handled by others, such as transporting patients, a task usually done by support staff, or providing additional emotional support, which would often be shared with family members.

Participants expressed a complex mix of understanding and resentment regarding RNs leaving to work as travelers for better pay. However, they also acknowledged travel RNs as a valuable resource for addressing staffing issues and wished that their units had brought them on sooner. Additionally, strong RN-to-patient ratios were seen as a positive, and access to experienced staff was deemed critical.

#### Personal protective equipment and safety.

Safety refers to the presence and availability of resources, processes, or systems that protect RNs from physical, emotional, or occupational harm while performing their duties. This includes, but is not limited to, the provision of adequate PPE, adherence to infection control policies (e.g., vaccination mandates, symptom checks), and access to security personnel. PPE and vaccines were the most spoken of issues around safety.

Some healthcare workers felt unsafe due to inconsistent safety protocols, particularly before all patients were swabbed for COVID-19. They preferred working in COVID-19 units where full PPE was standard because on other acute units, there was uncertainty about whether patients had the virus and subsequently less PPE. The initial lack of clear separation between COVID-19 and non-COVID-19 units heightened anxiety. Although safety improved after units were assigned, frustration persisted with coworkers who did not believe in vaccines and patients who frequently removed their masks, contributing to a sense of inevitability about getting COVID.

Many healthcare workers expressed confidence and a sense of safety in their workplace due to the availability of proper PPE. While masks were mandated in the hospital, but not always outside, staff felt safer in the COVID-19 unit because of the full protective gear required there. This reassurance of safety allowed them to focus on their duties and manage the stress of the pandemic more effectively, knowing they were well-protected.

#### Benefits, compensation, and gifts.

Participants expressed appreciation for certain benefits and suggested additional financial incentives and support to make working more appealing or manageable. These included hazard pay, tuition reimbursement, parking, accessible childcare, and benefits such as paid time off (PTO), Family Medical Leave Act (FMLA) leave, and disability support. Additionally, they appreciated small tokens, cards, food, and parties from the community, managers, hospital, and others to show appreciation and recognition, including ice cream or pizza parties.

Participants expressed dissatisfaction with the access to childcare assistance and raised concerns about the fairness and transparency of available benefits and compensation. Additionally, some participants felt frustrated with the generic nature of gifts from hospital leadership, feeling that small gifts like ice cream or a pizza party would not solve their problems.

Participants expressed their gratitude for the available benefits and compensation. In one instance, a colleague donated PTO to support a team member during a family emergency, which was deeply appreciated. The act of receiving gifts made participants feel valued and acknowledged, particularly when the gesture came from an individual.

#### Communication and knowledge.

During COVID-19, participants noted that the protocols and knowledge they needed were rapidly changing due to the new and emerging disease. Some participants felt they lacked access to essential information, highlighting the necessity for improved communication channels. Participants expressed the need for greater communication and transparency regarding rapidly changing protocols and knowledge during the COVID-19 pandemic to ensure they could stay up to date on best practices. Staff recommended improving communication channels by suggesting a variety of accessible and consistent methods to help RNs and NAs obtain the information they need. Participants noted that communication channels such as town hall meetings, Webex staff meetings, training sessions, journal clubs, and the intranet were valuable tools for obtaining essential information. Transparent communication and routine check-ins, including access to a resource RN, were highlighted as crucial for ensuring all staff were well-informed.

#### Health, wellbeing and stress management.

Participants discussed how hospitals provided health, well-being, and stress management resources such as on-site cafeterias and coffee, opening conference rooms for lunch, providing break/quiet rooms, offering pet therapy, having TVs available, hosting support groups, offering Employee Assistance Programs (EAP), utilizing wellbeing indexes, providing online resources for wellness and meditations, and encouraging physical activity. These resources can help take care of basic needs, manage stress, cope with mental health challenges, and cope with workplace stressors.

Participants highlighted the need for more time and support to attend to their basic needs, such as having proper lunch breaks and staying hydrated. While the hospital promoted online mental health and stress management services, very few participants interviewed actually described utilizing these resources. It is important to note how the availability of these virtual resources did not necessarily translate to the RNs feeling like they had the time to access them, especially if they were not even able to take regular lunch breaks.

Several participants talked about using therapy offered through EAP and found it helpful. One participant mentioned that they would not have sought therapy if they had not known about EAP. The resource helped them cope with the grief and loss they were experiencing outside of work, enabling them to continue performing well in their job despite the personal challenges they were facing.

## Discussion

At present, the start of the COVID-19 pandemic was nearly six years ago. While this research was framed as highlighting gaps in the ability to respond to a pandemic, it also illuminates broader systemic weaknesses in healthcare’s ability to manage crises and maintain quality care in routine practice. The findings provide insight into the experiences of RNs and NAs, identifying stressors, available resources, and gaps in support systems. Addressing these gaps is critical for improving workforce well-being and strengthening crisis preparedness. In light of these findings, this discussion will outline the major themes, strengths and limitations of the study and offer recommendations for future research. Additionally, it will provide actionable suggestions for RN managers and health system leaders to translate these insights into meaningful policy and practice improvements.

One key theme emerging from this study is the disproportionate exposure of RNs to COVID-19, leading to significant stress and burnout. RNs spend the majority of patient contact time, accounting for 86.1% to 88.2% of total time in intensive care unit patient rooms, compared to physicians’ 9.9% to 13.1% [3]. This prolonged exposure translated into higher mortality rates among RNs than physicians [4]. Given their increased mortality rates, low-paid health workers, such as RNs and nursing home staff, were referred to as the “sacrificial lambs” of the pandemic in some reports, [32] reinforcing the essential role of nursing in crisis response and the necessity of ensuring their protection and support. Data from the National Nurses United revealed significant racial disparities in COVID-related RN deaths, with Black and Filipino nurses disproportionately affected, underscoring critical equity issues that require further exploration to address systemic inequities and ensure protection for all nurses [33]. Past research has extensively linked workplace stressors to burnout and its negative consequences for patient care and RN retention [9,12,34]. However, while studies have quantified the financial and organizational impacts of turnover, [35,36] there remains a gap in understanding the specific stressors faced by RNs and NAs during the COVID-19 pandemic and how they perceived available resources [23,24].

Another critical finding was the importance of authentic and RN-centered resource delivery. Participants perceived similar resources differently based on whether they felt the support was genuine or superficial. Unit managers were identified as crucial sources of support, as their presence was seen as personal and meaningful, contrasting with impersonal gestures from hospital leadership. Research supports the role of authentic leadership in fostering trust, job satisfaction, and retention [37,38]. In addition to nurse managers, chaplains were seen as a significant source of support for nursing staff, particularly because the chaplains proactively came to them making this support more accessible. Research on chaplain care for healthcare staff continues to expand, underscoring the vital role chaplains play in addressing workplace challenges like work-life stress, moral distress, and ethical concerns. Interventions among pediatric ICU nurses demonstrate improved coping, enhanced work-life balance, and greater optimism and problem-solving abilities [39]. Our findings are in alignment with other recent studies which highlight their significant contributions during the heightened stress of the COVID-19 pandemic, where chaplains provided essential support to staff [40–42]. This study contributes to the growing evidence that chaplains may be especially suited for supporting nursing staff because they can deliver support in an authentic and nurse-centered way. Furthermore, embedding support into nursing workflows, including compensated educational time, unit managers collaborating with night and weekend teams, and guaranteeing resource accessibility, was deemed vital for effectiveness.

Participants also reported that rapidly changing policies and procedures were major stressors and that nurse managers helped communicate these changes. Nurse managers serve as intermediaries between administration and front line staff, and their ability to effectively communicate and implement policy changes is crucial [43]. However, during the pandemic, nurse managers faced extreme workloads, ethical dilemmas, and high stress, requiring both individual and structural interventions to support their role [44–46]. Research indicates that leadership styles such as transformational, authentic, ethical, and servant leadership positively influence RN well-being [38]. Thus, strengthening RN manager competence and job satisfaction through targeted interventions could yield downstream benefits, including improved care quality and reduced turnover [47–51].

Staffing shortages exacerbated stress and burnout, leading to increased turnover and loss of institutional knowledge as experienced staff retired early. Participants expressed frustration over being reassigned to unfamiliar units without adequate support or cross-training, which added to their stress. Workplace relationships were related to higher stress levels, with participants reporting that coworker support, or lack thereof, affected their ability to cope. Bullying, racism, and ineffective communication further compounded stress, underscoring that adequate staffing alone does not resolve workplace challenges. State-mandated RN staffing ratios have been shown to improve patient outcomes, reduce burnout, and increase job satisfaction [52]. Additionally, while travel RNs do not negatively impact patient outcomes, they are often viewed unfavorably by permanent staff, complicating team dynamics [53]. Increased wages do not directly reduce burnout but do improve job satisfaction and retention [54]. These findings highlight the need for holistic staffing strategies that go beyond hiring additional personnel to address underlying workplace culture and support structures.

### Strengths and limitations of the work

The study has some limitations. The reliance on retrospective interviews, conducted in 2021 after COVID-19 vaccines became available, introduces the potential for recall bias, as participants may not accurately remember or may reinterpret past experiences. Additionally, given the extended data collection period, it is challenging to distinguish between appraisal and existing coping or adaptation, particularly as participants expressing positive (non-threat) appraisals may reflect post-coping adaptations rather than initial appraisals of COVID-related stressors. A limitation of the study was that transcripts were not returned to participants for review, and member checking was not conducted; however, the results were disseminated back to participants to disseminate findings. The findings may also have limited transferability, as data were collected from a single hospital, restricting applicability to other healthcare settings.

Nursing assistants, particularly those working on COVID floors, were harder to recruit than registered nurses, leading to their disproportionate representation in the study and potentially limiting insights into assistive personnel’s experiences during the pandemic. Further research could include comparative designs between RN and NA workplace stressors to further isolate and understand the experiences of NAs. Furthermore, the present analysis does not examine individual coping dimensions, which are a critical component of Lazarus and Folkman’s model as part of secondary appraisal. Future research may include individual coping to understand how nursing staff navigate stressors in addition to workplace resources. Additionally, despite reflexivity efforts, researcher bias remains a concern, as all study team members had backgrounds in nursing, which could have influenced data interpretation. To address these limitations, future studies could include multiple institutions, collect data closer to real-time to reduce recall bias, integrate individual coping experiences, and incorporate external researchers to enhance objectivity in qualitative analysis.

Despite these limitations, this study demonstrates several strengths, including its use of Lazarus and Folkman’s Stress and Coping Model, which provides a well-defined theoretical framework for understanding how RNs and NAs managed stress during the COVID-19 pandemic. This study’s strengths lie in its in-depth exploration of the multifaceted stressors faced by both RNs and NAs, categorizing them into personal, interpersonal, organizational, and societal domains. By including both positive and negative appraisals, the study provides a nuanced understanding of nursing staff’s RNs’ experiences during a crisis. The rigorous qualitative methodology, including directed content analysis, investigator triangulation, and an audit trail, enhances the credibility and dependability of the findings. Additionally, the purposeful stratified sampling ensured diverse representation of both COVID and non-COVID inpatient nursing staff, allowing for a more comprehensive exploration of their experiences.

### Recommendations for further research

Building on the findings of this study, several directions for future research are recommended. The findings underscore the urgent need for health systems to implement evidence-based strategies that enhance crisis preparedness and address long-standing workforce challenges. This study reveals a critical cross-cutting theme centered on the authentic and nursing-focused delivery of resources. Specifically, it identifies six key types of resources necessary for supporting healthcare workers: emotional support, staffing, safety, compensation, communication, and stress management. These insights should guide future research to prioritize the testing and development of targeted interventions aimed at boosting the resilience and well-being of healthcare workers, such as chaplain-led interventions. Future research should also incorporate individual coping dimensions to provide a more comprehensive understanding of how both personal and organizational factors influence stress and coping mechanisms. The COVID-19 pandemic has highlighted significant gaps in the ability of health systems to respond effectively to both crises and routine care needs. Addressing these deficiencies requires developing and evaluating RN-centered interventions that offer robust support mechanisms for RNs and NAs, focusing on stress management, resource availability, and overall workplace well-being. Additionally, future research could explore other operational outcomes such as staff burnout and staff intention to leave or turnover. Collaboration with NAs, RNs, nurse managers, health system leaders, and governmental entities will be crucial in designing interventions that are practical, scalable, and sustainable. By investing in comprehensive intervention development and testing, future research can play a pivotal role in preparing the nursing workforce for future crises and enhancing quality of care and organizational outcomes.

### Implications for policy and practice

Based on the current findings, several actionable strategies and recommendations can be implemented at the unit, organizational, and policy levels to enhance supportive workplace environments and address workplace stress during crises. At the unit level, recommendations include providing more resources and authentic leadership training for RN managers and ensuring management is physically present across all shifts to support staff [55–57]. Organizational-level recommendations focus on maintaining access to essential services during crises, offering mental health and wellness programs, enhancing communication systems, providing structured training, increasing compensation and benefits, sustaining a supportive workforce, and focusing on staff retention [18,55–65]. Societal and policy recommendations call for establishing state-mandated adequate RN-patient ratios and enforcing vaccine mandates to ensure workplace safety [66–69]. For a detailed summary of participant-driven recommendations and the supporting evidence base, refer to Table 4. By adopting these strategies, health systems can better support their workforce and improve overall care delivery in both routine and crisis situations.

**Table 4:** Recommendations for supportive nursing workplace environments during crisis.

Unit-Level Recommendations
1. Support RN managers with more resources and authentic leadership training as they may be able to help nursing staff in a way that feels more supportive (54)
2. Encourage management to be physically present across all shifts (e.g., night shift) to provide visibility and support and strengthen relationships with all staff (55, 56) 3. Increase the availability of chaplains to support staff during crises and consider implementing chaplain-led structured interventions (38, 40)
**Organizational-Level Recommendations**
1. Maintain access to essential services during crisis (e.g., coffee, food, break room) (57)
2. Offer access to mental health resources and wellness programs (18,58), such as therapy and gym memberships (59)
3. Enhance systems so that communication is consistent, transparent, accessible, and inclusive of all staff across shifts (e.g., RNs and NAs, day/night/weekends). If leadership provides opportunities to listen to staff concerns, follow up with actions taken (55–56)
4. Provide structured training opportunities when RNs are asked to perform new skills or take care of new patient population (e.g., PPE, floating to unfamiliar floors) (60, 61)
Increase compensation and benefits to reflect crisis-related demands (e.g., hazard pay, childcare, grocery shopping) (57, 62)
6. Sustain a supportive workforce (e.g., patient transportation, family relations, answering phone and call bell) to reduce additional work burdens that can fall on RNs (63)
7. Focus on staff retention to maintain experience and knowledge (64)
**Societal/Policy Recommendations**
1. Establish and enforce state-mandated adequate RN-patient ratios to ensure safe workloads (65, 66)
2. Enforce vaccine mandates to enhance workplace safety (67, 68)

## Conclusion

This qualitative descriptive study explored the experiences of RNs and NAs providing inpatient care during the COVID-19 pandemic, focusing on their work-related stress, how they appraised that stress, and the workplace resources available or needed to manage these challenges. The findings highlight critical gaps in crisis preparedness, leadership support, and staffing strategies. Addressing these issues is essential to improving RNs’ and NAs’ well-being and ensuring high-quality patient care. By investing in sustainable workforce solutions, fostering authentic leadership, and implementing meaningful systemic changes, health systems can create a healthcare environment that better supports and values its nursing workforce.

## Supporting information

S1 Appendix Interview guide. (DOCX)

S2 Appendix COREQ (Consolidated criteria for reporting qualitative research). (DOCX)
