# Supplementary material for: Experiences of registered nurses and nursing assistants during COVID-19: Work stress, stress appraisal, and workplace resources; A qualitative descriptive study
Source: PLoS One. 2026 Mar 27;21(3):e0345525. doi: 10.1371/journal.pone.0345525 (PMC13029790; doi:10.1371/journal.pone.0345525)
Supplement: S1 Appendix — (DOCX) [file pone.0345525.s001.docx]

**Supplemental File 2:** *Interview Guide*

COVID-19 Experiences and Perceptions

1. Tell me about how your work as a nurse/nursing assistant has changed since the COVID-19 pandemic.

Prompts: Changes from your work before the pandemic

1. patient population
2. work procedures
3. staffing
4. job requirements
5. In general, how have you felt about your job since the beginning of the COVID-19 pandemic?

Prompts:

1. Changes in how you feel about your job
2. Before vs. after the pandemic
3. Changes in feelings about job over the course of the pandemic (I.e. waves of the pandemic; early versus now)
4. How have you managed any additional stress or burden that you’ve experienced due to COVID-19?

1. What kinds of support has been offered through the hospital during COVID-19?

Prompts:

1. Support through the hospital?
2. Manager?
3. Co-workers?
4. Resources?
5. Follow up: Recommendations (i.e. What do you wish you had support for/resources for)?
6. Is there anything else related to how COVID-19 has affected you and your work that has not yet been addressed during the interview but that you would like to share?
